# Current understanding on the roles of gut microbiota in fish disease and immunity

**DOI:** 10.24272/j.issn.2095-8137.2018.069

**Published:** 2018-07-03

**Authors:** Jin-Bo Xiong, Li Nie, Jiong Chen

**Affiliations:** 1 Laboratory of Biochemistry and Molecular Biology, School of Marine Sciences, Ningbo University, Ningbo Zhejiang 315211, China; 2 Key Laboratory of Applied Marine Biotechnology of Ministry of Education, Ningbo University, Ningbo Zhejiang 315211, China

Intensive aquaculture has increased the severity and frequency of fish diseases. Given the functional importance of gut microbiota in various facets of host physiology, modulation of this microbiota is a feasible strategy to mitigate emerging diseases in aquaculture. To achieve this, a fundamental understanding of the interplay among fish health, microbiota, and invading pathogens is required. This commentary focuses on current knowledge regarding the associations between fish diseases, dysbiosis of gut microbiota, and immune responses. Furthermore, updated research on fish disease from an ecological perspective is discussed, including colonization resistance imposed by commensals and strategies used by pathogens to overcome resistance. We also propose several directions for future research, such as exploration of the causal links between fish diseases and specific taxa, and identification of universal gut microbial biomarkers for rapid disease diagnosis.

Fish aquaculture is the fastest growing animal food sector to support the growing human population, with a year-on-year growth rate of 10.4% (FAO, 2013). However, fish production is threatened by numerous diseases (Lafferty et al., 2015). This is particularly pertinent to aquacultural systems that impose various stressors on aquatic animals (Lafferty et al., 2015; Li et al., 2017a). Traditionally, antibiotics have been widely applied to prevent and treat diseases in aquacultures. However, antibiotic abuse has been highlighted in the transfer of resistance genes among pathogens, and has raised concerns regarding environmental pollution and consumer safety (Brandt et al., 2015). In recent years, the introduction of probiotics has been considered a sustainable strategy to improve fish health and protect them from emerging diseases (de Bruijn et al., 2017). Despite the extensive list of candidate probiotics investigated in previous studies (Dawood et al., 2016; Liu et al., 2018; Ramesh et al., 2017), successful application has been limited, as reported in a survey of farmers (Xiong et al., 2016). The lack of consistency in probiotic performance may be due to unsuccessful colonization as a result of sudden changes in habitats, e.g., from aerobic culture conditions to the anaerobic intestines (Giatsis et al., 2016). In addition, the fish gut is a main pathogen transmission route and a portal of entry (de Bruijn et al., 2017; Li et al., 2017a; Ringø et al., 2007; Zhang et al., 2015). Therefore, understanding the factors that dictate the invasion of pathogens and establishment of probiotics in the intestine will provide an initial step towards predicting and treating fish diseases.

Gut microbiota can affect fish physiology, development, life span, immunity, and barriers against pathogens (Burns et al., 2016; Nie et al., 2017; Smith et al., 2017; Yan et al., 2016). Therefore, the gut microbiota plays an indispensable role in fish fitness. Several recent reviews have centered on the diversity and functions of bacterial communities in healthy fish (de Bruijn et al., 2017), as well as on the external factors that affect fish gut microbiota (Wang et al., 2017) and interactions between gut microbiota and innate immunity in fish (Gómez & Balcázar, 2008; Nie et al., 2017). However, most previous studies have focused on factors that govern healthy gut microbiota, such as diet, rearing conditions, and fish genotype (Schmidt et al., 2015; Sullam et al., 2012; Yan et al., 2016). In contrast, few studies have reported on the interplay among gut microbiota, fish immunity, and disease (Nie et al., 2017). In this commentary, we summarize current knowledge on the associations between fish immunity, gut microbiota, and invading intestinal pathogens. We also highlight recent progress in uncovering the ecological processes of fish diseases.

According to the diversity resistance hypothesis, a more diverse microbial community harbors greater probability of having a species with an antagonistic trait toward an invader or pathogen (Fargione & Tilman, 2005). Consistent with this assertion, higher alpha diversity (mean species diversity at the habitat level) is frequently detected in healthy fish compared with diseased fish, such as largemouth bronze gudgeon (*Coreius guichenoti*) (Li et al., 2016), crucian carp (*Carassius auratus*) (Li et al., 2017a), and ayu (*Plecoglossus altivelis*) (Nie et al., 2017). One possible explanation for this pattern is that the invading pathogens out-compete the gut commensals, thereby reducing diversity. Similarly, gnotobiotic zebrafish (*Danio rerio* Hamilton, 1822) have been shown to be more sensitive to pathogenic infections (Oyarbide et al., 2015). In addition, antibiotic administration generally reduces diversity of the gut microbiota, which, in turn, facilitates colonization by external pathogens (He et al., 2017). Indeed, gut microbial diversity has been used as a biomarker of fish health and metabolic capacity (Clarke et al., 2014), with low diversity and stability of the microbiota closely associated with fish disease (He et al., 2017; Li et al., 2017a; Nie et al., 2017). A preponderance of evidence has demonstrated that more diverse gut communities exert greater protective effects on the host (De Schryver & Vadstein, 2014; Johnson et al., 2008; Zhu et al., 2016). In this regard, gut microbial diversity in fish should be maximized to reduce pathogenic invasions in aquaculture systems.

Fish are in continual contact with a complex and dynamic planktonic microbiota. Therefore, it is expected that gut microbiota in fish will be largely affected by microbes in the environment. This has been demonstrated by the high similarity between water and gut microbiotas of Atlantic cod larvae (*Gadus morhua*) (Bakke et al., 2013), rainbow trout (*Oncorhynchus mykiss*) (Wong et al., 2013), and tilapia larvae (Giatsis et al., 2015). Based on the co-evolution theory, however, to improve host fitness, mutualistic relationships between fish and gut microbiota should be tightly regulated to ensure suitable bacterial colonization (McFall-Ngai et al., 2013). As a result, gut bacterial communities between recently caught and domesticated fish share similar community structures (Roeselers et al., 2011). Intriguingly, reciprocal gut microbiota transplants between zebrafish and mice have shown that the relative abundance of lineages changes to resemble normal gut microbiota of the recipient host (Rawls et al., 2006). Similarly, previous meta-analysis has revealed that host phylogeny determines the composition of fish gut bacteria, even at the bacterial phylum level (Sullam et al., 2012). For example, the gut microbiota of largemouth bronze gudgeon is dominated by phyla *Proteobacteria*, *Actinobacteria*, and *Tenericutes* (Li et al., 2016), whereas *Gammaproteobacteria*, *Alphaproteobacteria*, *Firmicutes*, and *Bacteroides* are predominant in the gut of ayu (Nie et al., 2017). This pattern also holds true for different fish species (herbivorous *Ctenopharyngodon idellus*, carnivorous *Siniperca chuatsi*, and *Silurus meridionalis*) reared in the same pond (Yan et al., 2016). Indeed, it has been suggested that gut microbiotas of fish are distinct from those in rearing water and/or sediment (Li et al., 2017a; Schmidt et al., 2015; Zhang et al., 2018). However, this does not mean that the gut microbiota is temporally stable during the entire lifetime of the fish; rather, gut bacterial communities vary significantly during the developmental stages in healthy fish (Li et al., 2017b; Stephens et al., 2016; Yan et al., 2016; Zhang et al., 2018). This high temporal pattern is largely contributed to by maturation of the host (Burns et al., 2016; Zhang et al., 2018) as selection of gut microbiota is reinforced with time. Intriguingly, several species of fish exhibit core gut microbiota, including zebrafish (Roeselers et al., 2011), rainbow trout (Wong et al., 2013), channel catfish (*Ictalurus punctatus*), largemouth bass (*Micropterus salmoides*), and bluegill (*Lepomis macrochirus*) (Larsen et al., 2014), though location-dependent variations in gut microbiota also exist. These core lineages may be used as baselines for future probiotic trials.

It is worth emphasizing, however, that the tight link between fish and their gut microbiota can be disrupted by diverse variables, with host disease being the primary factor (Li et al., 2017a; Nie et al., 2017). Gut bacteria reside on mucosal surfaces, which provide the first line of defense against pathogens. Specifically, commensal bacteria compete for or modify the ecological niche and available nutrients to inhibit the colonization and proliferation of incoming pathogens in the intestine (Kamada et al., 2013). For example, well-known probiotic *Bifidobacterium* prevents pathogenic *Escherichia coli* invasion via acidification of the intestinal environment (interspecies barrier effect) (Fukuda et al., 2012). In addition, gut commensals can produce bacteriocins and proteinaceous toxins that specifically inhibit members of the same or similar bacterial species (intraspecies barrier effect). Therefore, susceptibility to pathogenic infection seems to rely, at least in part, on the structure of the host’s gut microbial community (Galindo-Villegas et al., 2012; He et al., 2017). Indeed, dysbiosis in the gut microbiota is frequently associated with fish disease (He et al., 2017; Nie et al., 2017). However, it is currently unclear whether changes in the microbial community are a cause or consequence of these diseases.

Responses of a community to disturbance (e.g., disease) are not solely the sum of the traits of individual species but are also dependent on interspecies interactions (Faust & Raes, 2012; Zhu et al., 2016). Our recent work showed that pathogenic infections have a significant impact on the gut microbiota, with diseased ayu exhibiting less complex and diverse interspecies interactions (Nie et al., 2017). Indeed, interspecies interaction analysis has been applied to identify candidate pathogens and/or probiotics in gut diseases (Buffie et al., 2015; Dai et al., 2018). Furthermore, it is apparent that populations, not clones, are the causal agents of some aquaculture diseases (Hou et al., 2018; Lemire et al., 2015). This idea overturned the traditional view that only a pathogen and/or virulence gene result in disease (Falkow, 1988), and led to the ‘ecological Koch’s postulates’, which aims to untangle the interplay between host health, microbiota, invading pathogens, and diseases (Vonaesch et al., 2018). However, current understanding on the ecological processes that govern the gut microbiota in fish is still in its infancy, and no consensus has yet emerged. For example, it has been reported that the relative importance of determinism increases as zebrafish mature (Burns et al., 2016), whereas other studies have shown the opposite trend (Li et al., 2017b; Yan et al., 2016). Understanding the factors that govern the gut microbiota provides an initial step to establishing and maintaining a healthy fish microbiome (de Bruijn et al., 2017; De Schryver & Vadstein, 2014). In this regard, exploring the underlying mechanisms of fish diseases will provide an integrated approach to systems biology and ecology.

Going a further step, gut signatures can also be associated with fish diseases. For example, taxa affiliated with genera *Vibrio*, *Aeromonas*, and *Shewanella* are overrepresented in the gut microbiota of “red-operculum” disease in crucian carp, whereas *Cetobacterium* species are indicators of healthy fish (Li et al., 2017a). Similarly, *Aeromonas* is a biomarker for largemouth bronze gudgeon suffering from furunculosis (Li et al., 2016). This phenomenon suggests that certain gut microbial signatures are indicative of host health status irrespective of disease pathogeny, as has been demonstrated in human gut diseases (Mancabelli et al., 2017). Recent mechanistic studies suggest that the inflammatory host response produces reactive oxygen species, which facilitate a competitive advantage to facultative anaerobic lineages, such as *Aeromonas* (Winter & Bäumler, 2014). To date, however, surprisingly few studies have examined the association between disease severity and degree of dysbiosis in the gut microbiota during disease progression in fish. As a result, it is unclear whether the transition from healthy to diseased gut microbiota is gradient-like or a discrete process (Knights et al., 2014). If the transition is gradual, gut microbial signatures could serve as independent variables for predicting the incidence of fish disease, similar to that observed in shrimp diseases (Xiong et al., 2017; Xiong et al., 2018).

In addition to direct inhibition, the fish gut microbiota also plays critical roles in epithelial renewal and maturation, which, in turn, regulate immune responses (Gómez & Balcázar, 2008; Wang et al., 2017). Under normal conditions, goblet cells secrete mucus, which functions as a barrier to inhibit migration of microorganisms out of the intestinal lumen (Ringø et al., 2007). A mature gut mucosa is also essential for distinguishing pathogens from commensals through pattern recognition receptors (PRRs, such as toll-like receptors, RIG-I-like receptors, NOD-like receptors and AIM2-like receptors), which detect bacterial antigens and activate signaling cascades to regulate immune responses (cytokines) (Pérez et al., 2010). For example, the toll-like receptor family, a representative member of PRRs, recognizes conserved structures in pathogens, which can recruit and regulate the immune and inflammatory cells that initiate and mediate systemic immune responses (Fasano & Sheadonohue, 2005). Additionally, commensals can protect the host by depriving invading pathogens of nutrients, secreting a range of antimicrobial substances and occupying the niche (de Bruijn et al., 2017; Gómez & Balcázar, 2008; Pérez et al., 2010). However, if this balance is disrupted, such as during pathogenic infections, the innate and adaptive immune systems are activated to prevent disease exacerbation. Conversely, there is a correlation between colonization of probiotics and innate immune responses, such as phagocytic and alternative complement pathway activities, which protect fish against pathogens (Balcázar et al., 2007; Kim & Austin, 2006).

Studies on gnotobiotic zebrafish demonstrate that the gut microbiota enhances the stability of β-catenin via activation of Wnt signaling, thereby promoting intestinal cell proliferation over normal ontogenesis (Cheesman et al., 2011; Rawls et al., 2006). Compared with germ-free zebrafish, conventionally raised zebrafish exhibit a greater abundance of genes associated with epithelial proliferation and innate immune response (Rawls et al., 2004). However, germ-free zebrafish with a commensal microbiota can robustly activate NF-κB and its target genes in intestinal and extra-intestinal tissues (Kanther et al., 2011). Similarly, colonization of commensals in larvae stimulates neutrophils and activates pro-inflammatory genes through the TLR/MyD88 signaling pathway and phagocytes, which can enhance disease resistance in zebrafish (Galindo-Villegas et al., 2012). Specifically, the gut microbiota induces intestinal macrophages by upregulating pro-IL-1β. The mature form of IL-1β (activated by pathogen infection) recruits neutrophils, thereby priming macrophages to eradicate pathogens (Kamada et al., 2013). Significant association between the gut microbiota and transcription level of secreted immunoglobulin M (sIgM, a proxy for adaptive immune development) has been reported during healthy zebrafish development (Stephens et al., 2016). Compared with functional B- and T-cell receptor immune-deficient zebrafish, wild-type zebrafish exhibit an individualized gut microbiota and increased determinism of gut microbiota assembly (Stagaman et al., 2017). Our recent work also showed pro-inflammatory cytokines IL-1β and TNF-α to be activated in response to pathogenic infections in ayu (Nie et al., 2017). On the other hand, administration of probiotics to sea bass (*Dicentrarchus labrax* L.) results in the downregulation of IL-1β and transforming growth factor-β (Picchietti et al., 2008). Collectively, these results indicate a normal gut microbiota contributes indispensable roles in regulating the fish immune system, and vice versa.

As described above, the host and gut microbiota have co-evolved multiple strategies to not only prevent colonization by external pathogens, but also suppress resident pathogens. However, pathogens have developed various strategies to overcome these barriers, including entry into the host, occupation of a unique niche, circumvention of commensals and host defense barriers, and acquisition of nutrients from fish hosts (Ringø et al., 2007). Specifically, pathogens express sortases and adhesins for anchoring to host intestinal cells. After attachment to the intestinal tract, pathogens produce toxins and hemolysins to aggressively damage the intestinal lining and induce inflammatory responses (Mazmanian et al., 2001; Ringø et al., 2007). There is evidence that the inflamed environment induces production of reactive oxygen and/or nitrogen species by the host, resulting in a bloom of facultative anaerobic bacteria (e.g., *Proteobacteria*) and reduction in obligate anaerobic bacteria (Winter & Bäumler, 2014). This shift in community composition compromises colonization resistance imposed by gut microbiota, thereby facilitating the overgrowth of potentially harmful indigenous bacterial species (Galindo-Villegas et al., 2012; He et al., 2017). To escape from host immune clearance, some enteric pathogens harbor a modified form of siderophore (chelating iron under iron-limiting conditions) that is not inhibited by host cell-secreted lipocalin 2, which can further promote the growth of pathogens (Fischbach et al., 2006). Additionally, pathogenic capsules promote virulence by reducing host immune responses (Singh et al., 2011). Gram-negative pathogens commonly encode the type 6 secretion system (T6SS), which enables pathogens to attack the resident microbiota and to confer them with a competitive advantage (Russell et al., 2014; Vonaesch et al., 2018). In addition, to counteract nutritional competition by commensals, some pathogens can use alternative or pathogen-specific nutrients, which expand the nutrient niche for their colonization (Fabich et al., 2008). Alternatively, invaders can also occupy a distinct niche during replication to reduce competition with commensals. For example, pathogenic *Citrobacter rodentium* expresses intimin, which enables its localization to the intestinal epithelial surface, where commensals do not normally occur (Kamada et al., 2012). Intriguingly, pathogens can sense cues (e.g., bile acids, temperature, and nutrient availability) from their host to regulate virulence genes at the appropriate location (Fraser & Brown, 2017; Vonaesch et al., 2018). This regulatory mechanism can therefore maximize the chance of successful invasion.

Once a pathogen escapes colonization resistance imposed by gut commensals and host immunity, it can replicate and further express diverse virulence factors to attack fish and cause disease. There is increasing evidence that pathogenic infections cause profound disturbances to the fish gut microbiota and immune responses (He et al., 2017; Nie et al., 2017; Ringø et al., 2007). Notably, variations in the gut microbiota of ayu are significantly associated with TNF-α and IL-1β expression levels (Nie et al., 2017). Similarly, antibiotic administration can also cause imbalance in the gut microbiota of zebrafish, resulting in a compromised immune response, which further increases susceptibility to infections (He et al., 2017). Molecular experiments further suggest that decreased water quality can promote pathogen virulence (Penttinen et al., 2016). Therefore, disease onset in fish can be attributed to a variety of disturbances, such as environmental stress and antibiotic administration, which disrupt the gut microbiota in stressed fish and enhance the virulence of pathogens.

In summary, the introduction of pathogens into hosts is antagonized by environmental pressure, fish filtering, and colonization resistance of gut commensals (Mallon et al., 2015). In healthy fish, the gut microbiota directly antagonizes the colonization or overgrowth of pathogens (Nie et al., 2017). These effects include competition for resources, niche exclusion, and suppression of virulence factors. In addition, pathogens are suppressed by immune clearance. In diseased fish, balances in the protective commensal microbial community and host immunity are disturbed by external factors. For example, antibiotic usage can decrease species diversity and alter gut microbial community structure in fish (He et al., 2017). Pathogenic infections have been shown to significantly disrupt interspecies interactions in the fish gut microbiota (Nie et al., 2017). These alterations may open up ecological niches for pathogenic invasions. Furthermore, environmental stresses may impose additional pressure on fish, leading to compromised immunity. Lastly, the expression of virulence genes in pathogens can also be induced by poor water quality (Penttinen et al., 2016; Zhou et al., 2012). These detrimental effects cumulatively attenuate resistance to colonization by pathogens and allow overgrowth of harmful colonies that may lead to disease.

Given the functional importance of the gut microbiota in improving host fitness, introduction or augmentation of beneficial microbes may be a promising approach for protecting fish from emerging diseases (de Bruijn et al., 2017). However, various studies have identified long lists of implicated microbes that may contribute to the gut microbiota dysbiosis-disease relationship, and these associations may reflect biomarkers of disease. Therefore, future work is required to explore the causal links between fish disease and specific taxa, which may enable us to optimize gut microbiota composition to mitigate fish disease. Pathogenic infections involve several phases: introduction, establishment, spread, and impact, which are governed by the environment, host, and gut microbiota (Mallon et al., 2015). To understand the mechanisms underlying fish disease, one should focus on the infection process from an ecological prospective (De Schryver & Vadstein, 2014; Xiong et al., 2016) instead of isolating potential pathogens from diseased fish. Next generation sequencing has allowed the identification of universal gut microbial biomarkers (common features of affected individuals) in various fish diseases from different regions. Therefore, we recommend that relevant information should be deposited into a public database, which could enable convenient cross-disease comparisons. This would facilitate rapid diagnosis as well as promote prediction of the course and prognosis of disease.
